# On-road driving impairment following sleep deprivation differs according to age

**DOI:** 10.1038/s41598-021-99133-y

**Published:** 2021-11-03

**Authors:** Anna W. T. Cai, Jessica E. Manousakis, Bikram Singh, Jonny Kuo, Katherine J. Jeppe, Elly Francis-Pester, Brook Shiferaw, Caroline J. Beatty, Shantha M. W. Rajaratnam, Michael G. Lenné, Mark E. Howard, Clare Anderson

**Affiliations:** 1 Turner Institute for Brain and Mental Health, School of Psychological Sciences, Monash University, Clayton, VIC 3800 Australia; 2 Seeing Machines, Fyshwick, ACT 2609 Australia; 3 Institute for Breathing and Sleep, Austin Health, Heidelberg, VIC 3084 Australia

**Keywords:** Human behaviour, Circadian rhythms and sleep

## Abstract

Impaired driving performance due to sleep loss is a major contributor to motor-vehicle crashes, fatalities, and serious injuries. As on-road, fully-instrumented studies of drowsy driving have largely focused on young drivers, we examined the impact of sleep loss on driving performance and physiological drowsiness in both younger and older drivers of working age. Sixteen ‘younger’ adults (*M* = 24.3 ± 3.1 years [21–33 years], 9 males) and seventeen ‘older’ adults (*M* = 57.3 ± 5.2, [50–65 years], 9 males) undertook two 2 h drives on a closed-loop track in an instrumented vehicle with a qualified instructor following (i) 8 h sleep opportunity the night prior (well-rested), and (ii) after 29-h of total sleep deprivation (TSD). Following TSD, both age groups displayed increased subjective sleepiness and lane departures (*p* < 0.05), with younger drivers exhibiting 7.37 × more lane departures, and 11 × greater risk of near crash events following sleep loss. While older drivers exhibited a 3.5 × more lane departures following sleep loss (*p* = 0.008), they did not have a significant increase in near-crash events (3/34 drives). Compared to older adults, younger adults had 3.1 × more lane departures (*p* = < 0.001), and more near crash events (79% versus 21%, *p* = 0.007). Ocular measures of drowsiness, including blink duration, number of long eye closures and PERCLOS increased following sleep loss for younger adults only (*p* < 0.05). These results suggest that for older working-aged adults, driving impairments observed following sleep loss may not be due to falling asleep. Future work should examine whether this is attributed to other consequences of sleep loss, such as inattention or distraction from the road.

## Introduction

Globally, more than 1.35 million people are fatally injured in road traffic crashes, with an additional 20–50 million people sustaining non-fatal serious injuries^1^. Drowsiness, due to insufficient sleep or driving during the night-time hours, is a major contributing factor to motor vehicle crash risk^2,3^. In the United States, drowsiness was estimated to contribute to 21% of all fatal motor vehicle crashes and 13% of severe injury crashes. Similarly, in Australia, drowsiness is involved in an estimated 20% of all fatal crashes, and 30% of severe injury motor vehicle crashes^4,5^. Crashes caused by drowsiness are both highly identifiable and preventable. For instance, sixty-six percent of drivers report having driven while drowsy in the past 5 years, and 19% report experiencing near crashes due to drowsiness^6^. Younger drivers are overrepresented in drowsiness-related crashes^7–9^, such that 18–24 year old drivers are 14.2 times more likely to crash during the night-time hours or during early morning driving^10^. This enhanced vulnerability may be due to an increase in driving exposure during these times, and/or an increased vulnerability to sleep loss compared to older drivers. While laboratory-based studies have examined this increased vulnerability of younger individuals to sleep loss^11–13^, no study has examined age-related vulnerability to drowsy driving using real, on-road driving outcomes. No data therefore exists characterising and quantifying the impairment of younger drivers relative to older drivers, when well-rested and drowsy.

The effect of sleep loss on real on-road driving performance in younger drivers has been well-documented. During sleep loss, younger drivers exhibit greater physiological signs of drowsiness including increased slow eye movements^2^ and longer blink duration^14^. Moreover, sleep loss in these younger drivers is associated with increased lane departures and speed deviations, longer braking response times, and increased near-crash events^2,14–17^. Using a fully instrumented vehicle on a closed loop track, 67% of younger drivers experienced a near-crash event during a 2 h drive, following a single night without sleep. Driving impairment was also exacerbated by driving time, such that lane departures increased by 7% with every 5-min of driving^14^. Using the same on-road/track study design, driving performance in shift workers (aged 19–65 years) was significantly impaired following a night shift relative to a night of ‘normal’ sleep. Post-night shift drives had significantly more near-crash events (37.5% vs*.* 0%) or were terminated early for safety reasons (43.8% vs. 0%), and had more lane deviations and ocular indices of drowsiness^2^. As reflected in this study, younger and older individuals are approximately equally involved in shift work. For instance, in Australia 34.9% of shift workers are younger adults aged 20–34 years old, while 39.3% of shift workers are aged 55 years old and over^18^, and in the United States 16.6% are younger (aged 25–34 years old), while 12.8% are older (aged 55 years old and over)^19^. Understanding and quantifying the impact of sleep loss on driving performance and crash risk is equally important for both younger and older drivers, particularly those still involved in occupations involving sleep loss (e.g., shift work).

Relative to younger drivers however, there is a lack of research examining the effect of sleep loss on older drivers, particularly using real on-road driving outcomes. Laboratory studies suggest that compared to younger adults, older adults are resilient to the effects of sleep loss, as they show smaller performance decrements on sustained attention tasks^11–13^, and less physiological sleepiness as exhibited by slow eye movements^11^. Driving simulator studies have revealed similar results with older drivers showing less drowsiness-related driving impairment compared to younger drivers^20–22^. While older adults appear to better tolerate sleep deprivation^11^, it is important to note that that they are still impaired by sleep loss, relative to themselves when well-rested. For example, older drivers (aged 52–74 years) had significantly more lane departures and reported greater subjective sleepiness during a simulated drive following sleep restriction^20^, compared to when well-rested. Additionally, older adults (65–76 years) had a slower mean reaction time and a greater number of PVT lapses during sleep deprivation, relative to when well-rested^11^. As older drivers represent a significant proportion of road users (e.g., almost half of all road users are older than 50 years^23^), there is a critical need to extend these laboratory findings to an on-road driving environment to better understand and quantify the impact of sleep loss on real-driving outcomes, in both younger and older drivers in order to design appropriate interventions.

We therefore examined the impact of age on driving performance and physiological measures of drowsiness following a single night without sleep in younger and older drivers during an on-road driving task emulating a naturalistic driving environment. Taking into consideration road usage and likelihood of remaining awake overnight (e.g., shift work), we focussed our study on drivers aged 20–35 years (‘younger’) and those aged 50–65 years (‘older’). We hypothesised that younger adults would show greater driving impairment and physiological drowsiness following sleep loss compared to older adults. We also hypothesised that older adults would not be resilient to sleep loss but would instead show increased driving impairment and physiological drowsiness relative to when they were well-rested. To test these hypotheses, sixteen younger and seventeen older drivers were recruited to undertake two 2 h driving sessions around a closed track loop in an instrumented vehicle—a well-rested (WR) condition for one drive after a full night of sleep, and one following 29 h of total sleep deprivation (TSD).

## Results

Sixteen younger adults and seventeen older adults completed the study. Demographics for each group are shown in Table 1. As expected, the groups differed in age and years of driving, but was not significantly different in subjective sleep questionnaires, sex distribution and oxygen desaturation index (ODI). To ensure participants were well-rested prior to participating in each condition, they were required to maintain an 8:16 sleep/wake schedule in the week preceding the drive. As displayed in Table 2, participants maintained their 8 h time in bed prior to both visits. While older adults slept on average 46 min less than younger adults in the week prior to the TSD condition (*p* = 0.017), there was no difference in the WR condition (*p* = 0.918), nor for the night prior to the drive in either the sleep deprived (*p* = 0.171) or well-rested (*p* = 0.454) condition.

**Table 1:** Participant demographic summary (*N* = 33).

Demographics	Younger adults (*N* = 16)	Older adults (*N* = 17)	*p* value
Age (years)	24.26 (3.15)	57.31 (5.17)	
Sex (M:F)	9:7	9:8	0.867
Body Mass Index (kg/m^2^)	23.56 (3.28)	24.41 (2.74)	0.428
Driving experience (years)	5.59 (2.12)	38.12 (4.79)	< 0.001
Weekly driving (h)	11.97 (7.74)	8.41 (6.66)	0.099
PSQI (/21)	2.45 (1.62)	2.44 (1.63)	0.912
ESS (/24)	3.63 (2.03)	2.83 (2.01)	0.263
ISI (/63)	1.38 (1.41)	1.82 (1.78)	0.516
MEQ (/86)	38.68 (5.36)	42.05 (4.98)	0.071
ODI4% (events/h)	0.48 (0.50)	1.34 (1.51)	0.058

Mean (SD). *PSQI* Pittsburgh Sleep Quality Index, *ESS* Epworth Sleepiness Scale, *ISI* Insomnia Severity Index, *MEQ* Morningness–Eveningness Questionnaire, *ODI4%* Oxygen Desaturation Index with a 4% desaturation criteria.

**Table 2:** Sleep parameters in the week preceding the on-road track study for younger and older drivers.

Condition	Variable	Younger adults	Older adults	*p* value
Well rested	Time in bed (h)	8.17 (0.25)	8.02 (0.17)	0.081
	Total sleep time (h)	6.32 (1.03)	6.37 (0.90)	0.918
	SOL (min)	24.55 (19.77)	10.07 (10.25)	0.022
	Sleep efficiency (%)	77.61 (13.68)	79.39 (11.77)	0.715
	WASO (min)	68.85 (37.21)	78.05 (53.23)	0.601
Sleep deprived	Time in bed (h)	8.30 (0.75)	8.05 (0.28)	0.229
	Total sleep time (h)	7.15 (0.77)	6.42 (0.28)	0.017
	SOL (min)	15.56 (16.91)	19.24 (29.62)	0.677
	Sleep efficiency (%)	85.65 (5.85)	79.83 (9.42)	0.050
	WASO (min)	44.95 (15.39)	65.41 (36.16)	0.052

*SOL* sleep onset latency, *WASO* wake after sleep onset. Mean (SD) shown.

### Driving events

#### Lane deviations

**Figure 1:**
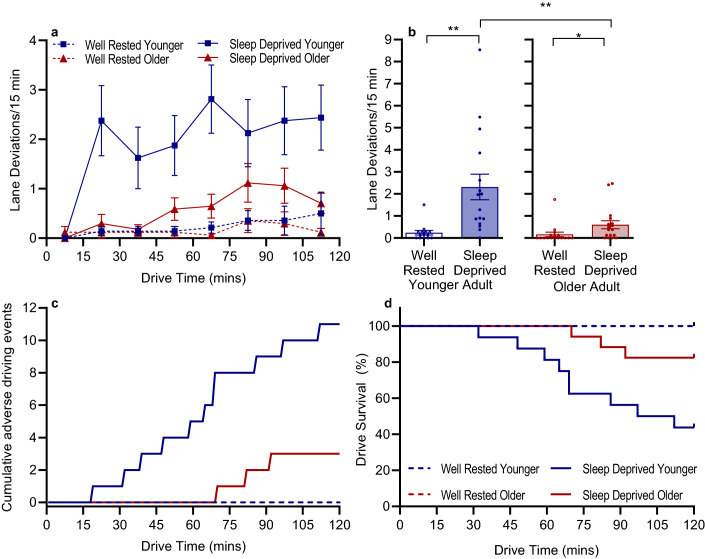
(**a**) Mean ± standard errors across driving duration by condition and age group (dashed line = well-rested; solid line = sleep deprived; blue = younger adults; red = older adults), for number of lane deviations per 15-min. (**b**) Individual and group data for lane deviations/15 min across the whole drive showing interactions between age and condition. (**c**) Cumulative histogram of 11 adverse driving events in the younger adult group (blue) and three adverse driving events in the older adult group (red) across time in each condition. (**d**) Kaplan–Meier survival curve of the nine drive terminations in the younger adult group and three drive terminations in the older adult group across time in each condition. Instructor interventions and drive terminations occurred only in the sleep-deprived drives. **p_adj_ < 0.001, *p_adj_ < 0.05. False discovery rate corrections were used.

Lane deviations are shown in Fig. 1a,b and Table 3. Following TSD, the rate of lane deviations per 15 min was  5.5 × greater than WR (95% CI 2.65, 11.38, *p* < 0.001) across both age groups. There was no significant effect of age (95% CI 0.16, 2.95 *p* = 0.113), however, sleep deprived younger drivers had 3.11 × more lane deviations relative to sleep deprived older drivers. While there was no significant interaction between age and condition (*p* = 0.278), this was likely due to both groups being impaired following TSD: younger adults had 7.37 × more lane deviations after TSD compared with WR (95% CI 2.80, 19.67, *p* < 0.001) whereas older adults had 3.5 × more than WR (95% CI 1.35, 8.97, *p* = 0.010). Adding time into the model found an effect of drive duration with the rate of lane deviations per 15 min increasing across the drive (*p* = 0.025). Results for three-way significant values (with time included) are shown in Supplementary Table 1, and all post-hoc comparisons are shown in Supplementary Table 2 and Supplementary Table 3 for younger and older drivers, respectively.

**Table 3:** Subjective sleepiness, ocular indices of drowsiness, and driving performance variables in well-rested and sleep-deprived condition, for younger and older drivers.

	Well rested		Sleep deprived		Significance values		
	Younger adults	Older adults	Younger adults	Older adults	Condition	Age	Condition × Age
KSS score	3.43 (1.57)	3.60 (1.72)	7.10 (1.74)*	6.55 (1.28)*	< 0.001	0.551	0.185
LFA score	4.48 (0.68)	4.74 (0.37)	3.19 (0.84)*	3.89 (0.85)*	< 0.001	0.001	0.144
Blink duration (ms)	163.21 (29.27)	195.47 (89.89)	240.66 (99.18)*	213.29 (119.75)	0.003	0.923	0.038
Blink rate (count/min)	15.19 (6.72)	13.36 (10.00)	17.04 (10.17)	13.21 (9.87)	0.551	0.268	0.360
LEC duration (ms)	1108.44 (323.47)	1041.26 (212.84)	963.47 (207.30)	1057.70 (203.00)	0.153	0.800	0.065
LEC rate (count/15 min)	4.43 (4.66)	5.05 (9.91)	14.63 (16.65)	7.20 (10.52)	0.007	0.096	0.076
PERCLOS (%)	3.17 (1.72)	3.51 (2.87)	5.48 (3.42)*	3.85 (2.66)	0.003	0.437	0.033
LD rate (count/15 min)	0.22 (0.38)	0.16 (0.42)	2.47 (2.31)*	0.60 (0.76)*	< 0.001	0.113	0.278
Near-crash events	0 (0%)	0 (0%)	10 (62.50%)*	3 (17.65%)*	< 0.001	0.013	n/a
Drive terminations	0 (0%)	0 (0%)	9 (56.25%)*	3 (17.65%)*	< 0.001	0.071	n/a

*Sig comparison compared to well-rested with an alpha of .01 or less. *n.s.* p > 0.1. Mean (SD) shown. *KSS* Karolinska Sleepiness Scale, *LFA* likelihood of falling asleep, *LEC* long eye closure, *PERCLOS* % time eyes are ≥ 80% closed in a minute, *LD* lane deviation, *n/a* no interaction examined with Fisher’s exact test.

#### Near crash events

A total of 13 out of 33 sleep deprived drives required the driving instructor to administer an emergency braking manoeuvre in response to a near-crash event to ensure the safety of the participant and study staff, compared with zero of the well-rested drives (Fisher’s exact test: *p* < 0.001), see Fig. 1c. There was an effect of age, whereby of the 14 total near crash events, 11 (79%) were from the younger drivers and 3 (21%) were from the older drivers (Fisher’s exact test: *p* = 0.013). There was an interaction effect of condition and age, as younger drivers had a greater amount (62.5%) of near crash events after TSD (Fisher’s exact test: *p* < 0.001). Whereas, older drivers were not impaired by TSD (Fisher’s exact test: *p* = 0.227), with 17.6% of older drivers with near crash events resulting in drive terminations.

#### Drive terminations

Twelve out of 33 TSD drives were prematurely terminated due to the driving instructor deeming the participant unable to maintain safe control of the vehicle, compared with zero of the WR drives (Fisher’s exact test: *p* < 0.001), with nine terminations (75%) from the younger group, and three (25%) from the older group (Fisher’s exact test: *p* = 0.071) See Fig. 1d. We also observed an interaction between condition and age, whereby drive terminations were significantly associated with condition for the younger drivers, but not older the older drivers, i.e., 56.25% of younger drivers did not complete the 2 h TSD drive (Fisher’s exact test: *p* = 0.001), compared to 17.6% of older drivers who did not complete the 2 h TSD drive (Fisher’s exact test: *p* = 0.227). The median drive time was 102 min for younger drivers, i.e. 50% of younger adults were deemed unsafe to drive after 102 min, while the median drive time for older drivers was 120 min (full drive).

### Ocular indices of alertness

#### Blink duration and blink rate

**Figure 2:**
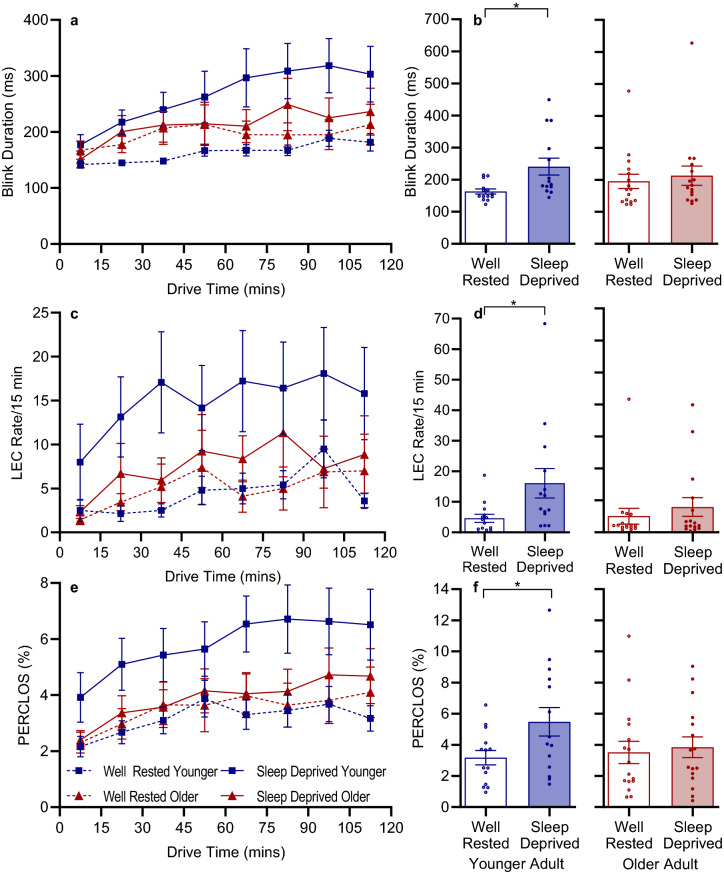
(**a**) Mean ± SEM across driving duration by condition and age for blink duration. (**b**) Individual and group data for blink duration across the whole drive showing interactions between age and condition. (**c**) Mean ± SEM across driving duration by condition and age for LEC/15 min. (**d**) Individual and group data for LEC/15 min across the whole drive showing age × condition interactions. (**e**) Mean ± SEM across driving duration by condition and age group for PERCLOS. (**f**) Individual and group data for PERCLOS across the whole drive showing age × condition interactions. Note: *LEC* long eye closure, *PERCLOS* % time eyes are ≥ 80% closed in a minute. *p_adj_ < 0.05. False discovery rate corrections were used.

Ocular outcomes are shown in Table 3 and Fig. 2. Blink duration was longer in the sleep-deprived drive compared to when well-rested (*F*_(1,28)_ = 11.03, *p* = 0.003). While there was no main effect of age (*F*_(1,28)_ = 0.01, *p* = 0.923), there was a significant interaction between age and condition (F_(1,28)_ = 4.74, *p* = 0.038), such that younger drivers had significantly longer blink duration following sleep deprivation (*p*_adj_ = 0.028), which was not evident in the older drivers (*p*_adj_ = 0.516) All other comparisons were non-significant (*p*_adj_ > 0.337). For the addition of time, blink duration also significantly increased with drive duration (*F*_(7,263.9)_ = 7.36, *p* < 0.001). See Supplementary Table 1 for three-way significance values, and Supplementary Table 4 for all post-hoc tests. Blink duration is shown in Fig. 2a,b.

For blink rate per minute, we observed no significant effect of condition (*F*_(1,28)_ = 0.10, *p* = 0.751), age (*F*_(1,28)_ = 1.33, *p* = 0.258) or condition by age interaction (*F*_(1,28)_ = 0.82, *p* = 0.314). When time was added to the model, we observed a main effect of drive duration for blink rate per minute (*F*_(7,188.7)_ = 5.24, *p* < 0.001). See Supplementary Table 1 for three-way interaction significance values and Supplementary Table 5 for drive duration post-hoc tests.

#### Long eye closure duration and rate

We classified long eye closures (LEC) as blinks > 500 ms. For LEC duration, there were no significant effects of condition (*F*_(1,28)_ = 2.16, *p* = 0.153), age (*F*_(1,28)_ = 0.07, *p* = 0.800), or condition × age interaction (*F*_(1,28)_ = 3.69, *p* = 0.065). When adding time into the model, there was a significant main effect of drive duration (*F*_(7,294.8)_ = 2.70, *p* = 0.010), such that the duration of LECs increased across the drive. See Supplementary Table 1 for three-way interaction significance values and Supplementary Table 6 for all post-hoc tests.

The number of LECs significantly increased in the TSD condition compared to the WR condition (*F*_(1,28)_ = 7.81, *p* = 0.007). There was no significant effect of age (*F*_(1,28)_ = 2.87, *p* = 0.096), and there was also no significant interaction between age and condition (*F*_(1,28)_ = 3.27, *p* = 0.076). Although almost reaching significance, our data suggest that younger adults exhibit more LEC following SD compared to themselves when well-rested, and compared to older adults when sleep deprived. When time was added to the model, LEC rate increased with drive duration (*F*_(7,237.7)_ = 4.92, *p* < 0.001). LEC rate per 15 min is shown in Fig. 2c,d. See Supplementary Table 1 for three-way interaction values and Supplementary Table 7 for post-hoc tests.

#### PERCLOS

PERCLOS was higher in the sleep-deprived condition compared to when well-rested (*F*_(1,28)_ = 10.89, *p* = 0.003).There was no significant effect of age (*F*_(1,28)_ = 0.62, *p* = 0.437), but there was a significant interaction between age and condition (*F*_(1,28)_ = 5.04, *p* = 0.033), such that younger adults had a significant increase in PERCLOS following sleep deprivation (*p*_adj_ = 0.011), which was not evident in the older adults (*p*_adj_ = 0.589) All other comparisons were non-significant (*p*_adj_ > 0.889). PERCLOS is shown in Fig. 2e,f. With time added into the model, PERCLOS increased with drive duration (*F* = 7.23, *p* < 0.001). See Supplementary Table 1 for three-way interaction significance values and Supplementary Table 8 for post-hoc tests.

### Subjective sleepiness and likelihood of falling asleep

**Figure 3:**
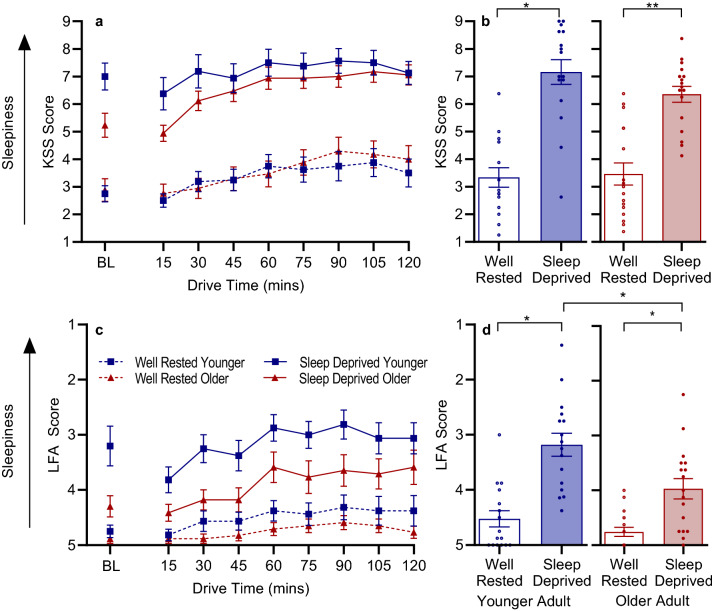
(**a**) Mean ± SEM across driving duration by condition and age group for KSS. (**b**) Individual and group data for KSS across the whole drive showing interactions between age and condition. (**c**) Mean ± SEM across driving duration by condition and age group for LFA. (**d**) Individual and group data for LFA across the whole drive showing interactions between age and condition. Note: dashed line = well-rested; solid line = sleep deprived; blue = younger adults; red = older adults). *KSS* Karolinska Sleepiness Scale, *LFA* likelihood of falling asleep. **p_adj_ < 0.001, *p_adj_ < 0.05. False discovery rate corrections were used.

Subjective sleepiness was measured pre-drive and every 15 min throughout the drive using the Karolinska Sleepiness Scale (KSS). KSS was significantly higher during the sleep-deprived drive compared to well-rested (*F*_(1,31)_ = 118.33, *p* < 0.001).There was no effect of age (*F*_(1,31)_, = 0.37, *p* = 0.551), nor any interaction between age and condition (*F*_(1,31)_, = 1.84, *p* = 0.185). KSS is shown in Table 3, Fig. 3a,b. With time added into the model, KSS exhibited an effect of drive duration (*F*_(1,143.9)_ = 7.38, *p* < 0.001), whereby KSS increased across the drive. See Supplementary Table 1 for three-way interaction significance values and Supplementary Table 9 for post-hoc tests.

Participants reported increased likelihood of falling asleep (LFA) in the sleep-deprived drive compared to when well-rested (*F*_(1,31)_ = 42.27, *p* < 0.001). There was a main effect of age, such that younger drivers reported greater LFA compared to older drivers (*F*_(1,31)_ = 11.94, *p* = 0.001). There was no interaction between age and condition (*F*_(1,31)_ = 2.19, *p* = 0.144). LFA can be seen Table 3 and Fig. 3c,d. When time was added to the model, a main effect of drive duration (*F*_(8,286.4)_ = 5.38, *p* < 0.001), with increasing likelihood of falling asleep across the drive. See Supplementary Table 1 for three-way interaction significance values and Supplementary Table 10 for post-hoc tests.

## Discussion

We found a high risk of near-crash events and/or hazardous driving behaviour during on-road driving in both younger (aged 21–33 years) and older (aged 50–65 years) drivers following one night of total sleep deprivation. Younger drivers had more than three times as many near-crash and lane deviation events than older drivers after sleep deprivation. Although both groups self-reported similar increases in subjective sleepiness and likelihood of falling asleep, younger drivers displayed greater objective sleepiness while driving as indicated ocular-based drowsiness indices. These findings suggest that greater vulnerability to the effects of sleep deprivation may contribute to the well-established increased risk of drowsiness-related road crashes in younger drivers.

Following sleep deprivation, driver safety was more compromised for younger drivers relative to older drivers, as reflected in both driving performance and physiological indices of sleepiness. Specifically, 62.5% of younger drivers had near crash events and 56% did not complete the 2-h drive following sleep loss, compared to 17.6% of older drivers. In addition, younger drivers had 3.5 × more lane departures, and increased blink duration and PERCLOS relative to older drivers following one night without sleep. This enhanced vulnerability to drowsiness in younger drivers is consistent with simulated driving outcomes, whereby younger adults had higher incidences of lane departures compared to older drivers, when sleep was restricted to 5 h^20^. This suggests that these observations are robust to methodological considerations, including study design and driver age (i.e., our ‘younger’ group is slightly younger, while our ‘older’ group is slightly younger).

We found that our older drivers had 2.5 × the number of lane departures and reported greater subjective sleepiness and increased likelihood of falling asleep when sleep deprived when compared with their well-rested state. Importantly, this has not been reported in previous simulator studies of age-related vulnerability to drowsy driving^24^, where the focus is on comparisons between younger versus older drivers, rather than the effects of sleep loss in older drivers, relative to themselves when well-rested. These findings have several important implications for older drivers who may drive following periods of sleep deprivation, e.g., commuting home after a night shift or night-time driving. Firstly, these observations likely apply to a significant number of road users. For instance, in the Unites States, more than half of all road users are aged 20–35 or 50–65 years (25.6% and 26.7% of all road users, respectively)^23^, which is similar to that reported in Australia (35.6% of road users are aged 20–40 years, and 30.8% are 50–70 years^25^). Moreover, the majority (> 70%) of individuals involved in shift work fall into these age categories (34.9% of Australian shift workers are 20–35 years, while 43.6% are 45–65 years)^18^. Secondly, any increase in driving impairment represents a greater risk for older drivers, particularly those older than 65 years. These drivers are at greater risk of fatal accidents per kilometre driven compared to all other drivers, except for young novice drivers^26^, and are more likely to sustain a fatal injury in a motor vehicle crash due to a pre-existing condition or frailty (playing a role in 50% of fatalities)^27^. As the risk of a sleep-related crashes is lower, yet the consequence is much higher, future work should examine the risk of adverse driving outcomes in these individuals. Thirdly, by 2050 the number of people older than 60 years is projected to double from 2015^28^, resulting in a greater number of older drivers on the roads. Strategies to reduce the number of motor vehicle crashes for older drivers, including targeting sleep, is therefore important. Fourthly, with normative ageing, sleep loss can interact with other factors such as sleep apnoea^29,30^, cognitive decline^31,32^, or the use of medications^33,34^, which in turn may further impact driving behaviour. While our study specifically examined the effect of sleep loss on driving performance in the absence of clinical impairment, the interaction between sleep loss and these comorbid factors warrants further investigation.

Despite ocular metrics being a strong indicator of drowsiness^35^, only the younger adults demonstrated increased ocular indices of drowsiness, including increased blink duration, LEC rate and PERCLOS during their sleep-deprived drive. Sleep loss related increases in ocular metrics have been well described in young drivers^14^, and are reliable and sensitive measures of drowsiness related driving impairment in younger drivers^36^. In contrast, in highly-controlled laboratory studies, older adults have shown a smaller increase in ocular ‘drowsy’ metrics and were less likely to fall asleep, consistent with our findings^11^. Given the notable shrinkage in sleep promoting systems as a function of age^37^, this finding is perhaps not unexpected. Despite this, our data and that of others, should not be interpreted as ‘immunity’ to drowsiness-related impairment; our older drivers had 2.5 × more lane departures following sleep loss, similar to a simulated driving study whereby older drivers performed worse when sleep restricted compared to when well-rested^20^. There are a number of interpretations for this observation. Firstly, large individual variability in ocular metrics^38^, may account for the lack of significant finding in this outcome. However, as individual variability is equally large for lane departures^38^, this may not adequately explain increased impairment, without comparable changes in eye closure parameters. Secondly, another form or marker of sleep-related impairment may be responsible for these adverse driving outcomes. Although speculative, one form of impairment may be enhanced distractibility. For instance, sleep loss is known to cause reduced inhibition^39,40^ and increased distractibility^41^, particularly while driving^42^, and older adults are vulnerable to this form of impairment^43^. Moreover, older drivers are more vulnerable than younger to driver distraction when well-rested^44^. It is therefore possible that this distractibility is exacerbated by sleep loss, explaining our observations; However, future work should systematically examine this.

Fourteen out of 33 (42%) sleep deprived drives required instructor intervention (due to an impending safety event), with 62.5% of younger adults and 17.6% of older adults experiencing a near-crash event. Overall, the proportion of drivers experiencing near-crash events was comparable to that observed in our previous study of night shift workers aged 19–65 years (42% and 37% respectively)^2^. The percentage of instructor interventions for younger sleep-deprived drivers was noticeably higher, however. We also found increased drowsiness with drive duration, which has been reported during on-road track studies^2^, on-road naturalistic night-time driving^45^, and simulated driving tasks^38^. For both younger and older drivers, subjective reports of sleepiness and likelihood of falling asleep started to increase between 15–30 min of driving, with ocular measures of drowsiness markedly increasing after around 30 min of driving. For younger drivers, near-crash events began to occur after 15 min of driving, while drive terminations began to occur 30 min into the drive, whereas for older drivers the first termination was 67 min into the drive. These data suggest that for younger adults, the deterioration in driving performance occurs earlier than our previous work in shift workers, for which critical driving events occurring after 45 min^2^. This may be due to a number of factors in our previous study of shift workers, including age (shift workers were, on average 48.7 years), sleep duration/time awake (shift workers obtained, on average 0.4 h of sleep), and time of day (morning drives compared to the ‘afternoon dip’). Our data therefore suggest that even shorter commutes of 15–30 min or more are likely to increase risk of drowsiness-related crashes in very drowsy younger drivers.

Our data should be interpreted with several caveats. Firstly, the study was conducted on a closed-circuit track free from a number of hazards and other environmental cues found in the real-world, such as vehicle and pedestrian traffic, billboard signs and advertisements, and rumble strips to which drivers would usually respond to and interact with. We note however, that the track used did include give way intersections, traffic lights, and railroad signs to mimic a naturalistic driving setting as closely as possible without risking driver and public safety; these features are typically not present in closed loop driving studies. Second, the presence of the study team and equipment may have increased driver alertness and awareness of alertness, compared to a drive without observation^2,14^. Third, the differential effect of sleep loss for older adults may be neurobiological in nature, or it may be due to their driving experience (e.g., spanning decades, rather than years). We are unable to disentangle these mechanisms, but these findings remain relevant to road users in terms of chronological age and experience. Fourth, as our drive sessions were scheduled by time since wake, our older group may have been tested at an earlier part of their biological day, further away in time from any circadian vulnerability in afternoon alertness (e.g., ‘post-lunch dip). However, as no differences were observed in the groups during the well-rested drives, and as circadian-related differences in performance between older and younger adults are less apparent in the first half the day^46^, (when our study was conducted), we suspect this impact to be minimal. Fifth, we did not include EEG data in our analysis, and so are unable to accurately determine the extent to which our drivers fell asleep at the wheel. Despite this, ocular parameters reflect fluctuating sleepiness levels due to central nervous system changes^47,48^, and are associated with poor driving outcomes^17^. Finally, participants were asked to report their level of sleepiness and likelihood of falling asleep every 15 min during the drive, which may have a brief alerting effect persisting up to 2 min^49^, and resulted in increased introspection. The driving session was also interrupted approximately every 30 min (a total of three times) for a short break of approximately one minute to administer a driver questionnaire. Despite these limitations, these confounds were consistent across both age groups and conditions, and the methodology we used is as close to real-life driving as is safely possible and provides the highest level of evidence for sleep-related driving impairment following total sleep loss. A major strength of our study is the use of highly advanced and validated eye tracking technology to monitor oculometrics throughout the drive. We were able to continuously monitor oculometrics throughout the drive, using Seeing Machines’ driver monitoring system (DMS; Seeing Machines, Canberra, Australia), a technology used in other naturalistic driving studies^50,51^. We also had strict eligibility criteria to ensure that those with health conditions that might impact sleep, such as sleep apnoea, were excluded from the study, ensuring any impairment was a direct result from sleep loss. Given the potential sex differences in response to sleep loss^52^, we also ensured our groups were balanced for sex. Future work should examine the extent to which this is also evident for driving impairment, and how this might interact with age.

Despite the increased risk of motor vehicle crashes associated with drowsiness, many drivers report driving when sleepy^6^. Using an instrumented vehicle around a closed track designed to mimic naturalistic driving, we provide critical evidence of age-related vulnerability to driving impairment following sleep loss. We replicate and expand previous studies showing the enhanced vulnerability of younger drivers, compared to older drivers. Importantly, we observed deterioration in driving performance and increased ocular measures of drowsiness after just 15 min of driving in younger drivers, following one night without sleep, indicating that even short commutes while sleep-deprived increases risk of a drowsiness-related motor vehicle crash. Although older adults better tolerated sleep deprivation while driving compared to the younger group, they still demonstrated drowsiness and driving impairment following sleep deprivation that could be further exacerbated by other health conditions that are more common in older adults (e.g. sleep apnoea). Given the lack of change in ocular measures (e.g., blink parameters, PERCLOS), it is likely that other aspects of sleep-related impairment play a causal role, and future research should examine alternative measures of drowsiness, such as distraction^41^ or altered gaze allocation^14^. With the growing number of ocular-based fatigue detection devices available, our data suggest that capturing both distraction and fall asleep events may benefit drivers of varying ages. Future research should focus on targeted intervention strategies (such as the graduated driver licensing law for with a night curfew introduced in Massachusetts, which led to a reduction in crashes for young drivers^53^) for these differentially vulnerable groups to help reduce the number of drowsiness-related motor vehicle crashes and overall road death toll^54^.

## Methodology

### Participants

Sixteen young adults aged 21–33 years (*M* = 24.26, *SD* = 3.14, 9 males) and seventeen older adults aged 50–65 years (*M* = 57.31, *SD* = 5.17, 9 males) were recruited through community advertising. Our sample represented ‘younger’ and ‘older’ adults of working age who were regular drivers and free from physical and cognitive decline. All participants held a valid Australian or international driver license for a minimum of three years and drove more than 100 km per week. Participants reported a habitual sleep duration of 7–9 h, habitual sleep times between 22:00 and 01:00 and wake times between 06:00 and 09:00 and did not nap more than once a week. Participants had no history of medical, psychiatric or sleep disorders, were not currently taking medication, did not have any visual impairment not corrected by lenses, were not colour blind, consumed less than 300 mg of caffeine a day and less than 14 standard alcoholic drinks per week, were non-smokers and non-shift workers, and had not travelled across two time zones in the past three months. Female participants were not pregnant, breastfeeding, or using hormonal contraception. Participants were screened for cardiovascular abnormalities with an electrocardiogram and were screened for obstructive sleep apnoea (defined as an ODI4% > 5) via one night of at-home respiratory monitoring using the ApneaLink (ResMed Corporation, Poway, CA, USA). Participants were screened for history of Axis 1 psychological symptoms or disorders with the Structured Clinical Interview for DSM-5^55^. Older adults completed a neuropsychological test battery consisting of the California Verbal Learning Test (CVLT)^56^, Comprehensive Trail Making Test (CTMT)^57^, WAIS-IV Digit Span^58^, National Adult Reading Test (NART)^59^ and Mini-Mental State Examination (MMSE)^60^ to ensure they were free from neurocognitive impairment, defined as scores ≤ 1.5 SD below age-matched norms for the CVLT, CTMT and WAIS-IV Digit Span, or a MMSE score of ≤ 24.

Participants provided written informed consent and were reimbursed for their time. The study was conducted in accordance with the Declaration of Helsinki and approved by the Monash University Human Research Ethics Committee.

### Procedure

Each participant underwent two 2 h driving sessions in an instrumented vehicle around a closed driving track (one after a night of normal sleep with 8 h time in bed, one following 29 h sleep deprivation) in a counterbalanced order, with a minimum one-week interval between sessions. To ensure that all participants were well-rested prior to admission to the study, participants maintained a self-selected fixed 8:16 h sleep:wake schedule for one week at home. Adherence was monitored by wrist actigraphy (Actiwatch 2, Phillips Respironics, USA), and time-stamped call-ins to the laboratory at bed and wake time. Participants were required to abstain from the consumption of caffeine, alcohol, nicotine, and over the counter, prescription or recreational drugs for 24 h prior to admission to the laboratory, confirmed by urine toxicology and a breathalyser test.

The night prior to the sleep-deprived driving session, participants stayed at the Monash University Sleep and Circadian Laboratory, Melbourne, where they were monitored by staff to ensure they remained awake. Participants were transported to the driving track and monitored en-route to ensure they remained awake during transport. The drive sessions began 5 h post-wake for the well-rested condition, and 29 h post-wake for the sleep-deprivation condition, beginning between 11:00 and 14:00. Drives times were the same within-participant (± 15 min) to control for any circadian variation in drive performance across the two conditions. All drives were completed in an automatic transmission Honda Jazz equipped with Seeing Machines’ driver monitoring system (DMS; Seeing Machines, Canberra, Australia), and a forward-facing camera (BlackVue, Pittasoft, Gyeonggi-do, Republic of Korea). A qualified driving instructor (R.W. or D.S.) who was blinded to the conditions of the study accompanied the participant to monitor safety and provide intervention (classified as a near-crash event) if there was a risk of collision, while a study technician sat in the back seat to regularly assess subjective sleepiness. Each drive was conducted on a closed track loop with both straight and curved sections, red light intersections, and a give-way turn. All participants completed the same driving route in both conditions. Prior to commencing the drives, participants were instructed to obey all speed limits, road signs, and follow all road rules, and then completed three practice laps to orient them to the track and study vehicle, before commencing the 2 h drive. Every 30 min of the drive, the participants were asked to put the car in park, and to complete the Sleepiness Symptoms Questionnaire. Driving sessions were completed in all weather conditions.

#### Driving impairment

A lane deviation was defined as at least two wheels crossing over the left or right lane markings^47^. Lane deviations were recorded by the driving instructor (R.W. or D.S.) during the drive. In addition, two scorers who were blinded to condition and age group (A.C. and B.S.) reviewed forward facing camera footage of each drive for lane deviations. A lane departure event was included when a lane departure was independently scored by two out of three scorers. An adverse driving event occurred when the instructor had to intervene to maintain control of the vehicle. Drives were terminated when the driving instructor deemed the participant too drowsy to maintain safe control of the vehicle.

#### Oculography

Ocular parameters such as eye-opening percentage were measured by monitoring eyelid and pupil movements via the Seeing Machines’ DMS. The driver facing camera was mounted to the top of the steering wheel mount, directly in front of the driver. The angle of the steering wheel mount was consistent throughout the study. Continuous eye-opening percentage was recorded throughout the entirety of the drive. As most eye tracking technologies are validated in young healthy populations^47,61,62^, it is unclear whether these technologies are appropriate for older adults, due to age-related ptosis (i.e., drooping of the upper eyelid) which will alter the eye aperture and potentially impact on blink calculations. Given this, we conducted extensive analysis and visual inspection of video footage to determine the ideal criterion to characterise blinks across our sample, and are confident our criteria and the criteria used in previous studies^38,63^ resulted in accurate blink characteristics for older adults. An eye blink was defined as the occurrence of an eye closing to below 50% (eye closing phase), followed by a closure to < 20% (eye closed), then reopening to at least 50% (reopening phase)^38^. The time (msec) from the eye closing phase to the eye-opening phase was taken as blink duration^63^. As well rested blinks are typically 100–500 ms^64^, we classified long eye closures (LECs) as any blinks greater than 500 ms in duration. Finally, the percentage of time the eyes were closed (< 20%) per minute (PERCLOS) was measured using the continuous eye-opening percentage^65–67^.

#### Subjective sleepiness

Participants verbally completed the Karolinska Sleepiness Scale (KSS) and the Likelihood of Falling Asleep Scale (LFA) prior to starting the drive (baseline), and every 15 min throughout the drive. The KSS requires participants to verbally rate their level of sleepiness in the past 5 min on a 9-point scale, ranging from 1—Extremely Alert, to 9—Extremely Sleepy^68^. The LFA requires participants to verbally rate the likelihood of them falling asleep in the next 5 min on a 5-point scale, ranging from 1—Very Likely, to 5—Very Unlikely^69^.

### Data analysis

We examined the impact of condition and age on driving performance, ocular measures of drowsiness, and subjective sleepiness in a two-drive condition (well rested; sleep deprived) by two-age group (young drivers; older drivers) model where participant was modelled as the random factor. We report the results of this 2 × 2 between-within subjects analysis, however, we have also repeated these analyses incorporating the effect of time on task. Time was treated as a discrete variable (eight by 15 min driving blocks). These three-way interaction results are reported in Supplementary Table 1. LFA ratings were transformed using a reflect and square root function (√k + 1 − n) to normalise data and improve negative skew. Blink duration and LEC rate was transformed using a logarithm function to normalise the data. PERCLOS and LEC duration were transformed using a square root function (√*n* + (√*n* + 1)) to normalise data.

#### Missing data

Some driving sessions were terminated early, resulting in missing data. This missing data is not random, as the drives were terminated when the instructor deemed the participant unable maintain control of the vehicle, which may yield a potential survivor effect. Due to this, missing data was imputed by using the last value carried forward technique (data for a missing 15-min driving block was replaced with data from the preceding 15-min block for that participant)^2,14^. This technique resulted in the conservation of 28 15-min blocks, ~ 6% of the total 480 observations (8-time bins × 30 participants × 2 conditions). This imputed data was then used to conduct additional analyses involving condition, age and time (Supplementary Table 1). In addition, forward facing camera footage was not collected for two drives, therefore lane deviations were scored for 64 out of 66 drives. Ocular data was not collected in *N* = 2 young adults, and *N* = 1 older adult, due to incorrect positioning of the driver or driving seat in relation to the driver facing camera resulting in the driver’s head and eyes being out of frame for the majority of the drive, or the camera tracking incorrectly onto the glare of the driver’s glasses. Therefore, all ocular data is reported for *N* = 14 young adults, and *N* = 16 older adults. Additionally, while the DMS allows for the collection of binocular data, monocular data was taken from the right eye only for *N* = 5 older adults, due to inaccurate tracking of the left eye, as verified by visual inspection of the driver facing footage.

### Statistical analysis

Raw data was processed using R (RStudio 1.1.463, Inc., Boston, MA, USA) to calculate the ocular parameters. All statistical analyses were conducted using SPSS 25 (IBM Corp, Armonk, NY).

Based on the observed mean differences in comparable study, which demonstrated medium effect sizes for lane departure (f = 0.36)^20^ outcomes when examining the interaction between sleep loss and age, we had 97% power to detect a medium effect size in lane departure outcomes with N = 33, using a between-within mixed model analysis. For ocular metrics, we used effect sizes from a laboratory-based study assessing the interaction between sleep loss and age, which demonstrated medium effect sizes for slow eye movements (f = 0.35)^11^. We had 97% power to detect a medium effect size in ocular outcomes with N = 33, using a between-within mixed model analysis.

To examine the effect of sleep loss and age on subjective sleepiness (KSS and LFA), and ocular measures of drowsiness, linear mixed model (LMM) analysis was used. Fixed effects of condition (well-rested and sleep-deprived) and age group (younger adults and older adults) were included in the model, including the interaction term. Participant was modelled as the random factor. For all LMM analyses, the covariance structure that yielded the lowest Schwarz Bayesian Criterion (BIC) was used^70^. For blink duration, blink rate, LEC duration, LEC rate, PERCLOS, KSS and LFA, a compound symmetry covariance structure was used. Post-hoc pairwise comparisons for interactions were conducted using a false discovery rate (FDR) comparison to control for familywise error^71,72^. Adjusted *p* values (*p*_adj_) are reported using the FDR “*q*” adjusted significance value for all post-hoc tests. As drive duration can interact with sleep loss, we added time into these models as supplementary data only (see Supplementary Table 1). For models including time, we used the last carried forward value method^2,14^ where the drive was terminated early.

Repeated measures Poisson regression was used to examine the effect of condition and age on rates of lane departure events. A contingency table analysis of Fisher’s exact test was used to examine the effect of condition and age group on near crash events and drive terminations. Kaplan–Meier survival curve was fitted to compare probability of drive survival in each age group and condition.

### Ethical approval

The study was conducted in accordance with the Declaration of Helsinki and approved by Monash University Human Research Ethics Committee.

## Supplementary Information

Supplementary Tables.
