# Supplementary material for: On-road driving impairment following sleep deprivation differs according to age
Source: Sci Rep. 2021 Nov 3;11:21561. doi: 10.1038/s41598-021-99133-y (PMC8566466; doi:10.1038/s41598-021-99133-y)
Supplement: Supplementary file 1 — Supplementary Tables. [file 41598_2021_99133_MOESM1_ESM.docx]

**On-road driving impairment following sleep deprivation differs according to age**

Anna W.T. Cai, Jessica E. Manousakis, Bikram Singh, Jonny Kuo, Katherine J. Jeppe, Elly Francis-Pester, Brook Shiferaw, Caroline J. Beatty^1^, Shantha M.W. Rajaratnam, Michael G. Lenné, Mark E. Howard, & Clare Anderson

**Supplementary Table 1. F and Significant values for the three-way mixed model analysis.**

|  | **F Value** | | | **Significance values For Condition*Age*Time** |
| --- | --- | --- | --- | --- |
| KSS score | 1.04 _(8,141.04)_ |  | | .41 |
| LFA score | 0.49 _(8,277.24)_ |  | | .86 |
| Blink duration (ms) | 0.73 _(7,234.20)_ | |  | .99 |
| Blink rate (count/min) | 0.93 _(7,173.13)_ |  | | .49 |
| LEC duration (ms) | 1.10 _(7,286.14)_ |  | | .37 |
| LEC rate (count/15min) | 1.06 _(7,214.13)_ |  | | .39 |
| PERCLOS (%) | 2.00 _(7,400.53)_ |  | | .054 |
| LD rate (count/15min) | N/A |  | | .44 |

|  | 30 mins | 45 mins | 60 mins | 75 mins | 90 mins | 105 mins | 120 mins |
| --- | --- | --- | --- | --- | --- | --- | --- |
| 15 mins | +0.53*  (0.17) | +0.44*  (0.16) | +0.61***  (0.16) | +0.86***  (0.20) | +0.92***  (0.21) | +0.95***  (0.21) | +0.86***  (0.21) |
| 30 mins | - | -0.08  (0.16) | +0.09  (0.14) | +0.34  (0.19) | +0.41  (0.21) | +0.44  (0.21) | +0.34  (0.23) |
| 45 mins |  | - | +0.17  (0.17) | +0.42  (0.21) | +0.48  (0.23) | +0.52  (0.23) | +0.42  (0.22) |
| 60 mins |  |  | - | +0.25  (0.15) | +0.31  (0.12) | +0.34  (0.16) | +0.25  (0.17) |
| 75 mins |  |  |  | - | +0.06  (0.18) | +0.10  (0.15) | +<0.001  (0.15) |
| 90 mins |  |  |  |  | - | +0.03  (0.10) | -0.06  (0.14) |
| 105 mins |  |  |  |  |  | - | +0.09  (0.10) |

**Supplementary Table 2.** Mean Difference (Standard Error) in Lane Deviations for Post-Hoc Comparisons for Time-On-Task in Younger Adults.

Note: ***= P_adj_ <.001, **= P_adj_ <.010; *= P_adj_ <.050

|  | 30 mins | 45 mins | 60 mins | 75 mins | 90 mins | 105 mins | 120 mins |
| --- | --- | --- | --- | --- | --- | --- | --- |
| 15 mins | +0.12  (0.16) | +0.06  (0.09) | +0.27  (0.10) | +0.27*  (0.09) | +0.65  (0.27) | +0.59*  (0.20) | +0.32*  (0.10) |
| 30 mins | - | -0.06  (0.14) | +0.15  (0.08) | +0.15  (0.16) | +0.53*  (0.19) | +0.47*  (0.18) | 0.47  (0.13) |
| 45 mins |  | - | +0.21  (0.16) | +0.21  (0.13) | +0.59  (0.27) | +0.53  (0.22) | +0.27  (0.11) |
| 60 mins |  |  | - | -<.001  (0.14) | +0.38  (0.17) | +0.32  (0.16) | +0.06  (0.12) |
| 75 mins |  |  |  | - | +0.38  (0.21) | +0.33  (0.13) | +0.06  (0.08) |
| 90 mins |  |  |  |  | - | -0.59  (0.12) | -0.32  (0.19) |
| 105 mins |  |  |  |  |  | - | -0.27  (0.14) |

**Supplementary Table 3.** Mean Difference (Standard Error) in Lane Deviations for Post-Hoc Comparisons for Time-On-Task in Older Adults.

Note: ***= P_adj_ <.001, **= P_adj_ <.010; *= P_adj_ <.050

|  | 30 mins | 45 mins | 60 mins | 75 mins | 90 mins | 105 mins | 120 mins |
| --- | --- | --- | --- | --- | --- | --- | --- |
| 15 mins | +0.14***  (0.03) | +0.20***  (0.04) | +0.24***  (0.04) | +0.26***  (0.05) | +0.30***  (0.05) | +0.32***  (0.05) | +0.31***  (0.05) |
| 30 mins | - | +0.06  (0.03) | +0.10*  (0.04) | +0.11*  (0.04) | +0.16**  (0.05) | +0.18**  (0.05) | +0.17**  (0.05) |
| 45 mins |  | - | +0.04  (0.03) | +0.05  (0.04) | +0.10  (0.04) | +0.12*  (0.05) | +0.11*  (0.05) |
| 60 mins |  |  | - | +0.02  (0.03) | +0.06  (0.04) | +0.08  (0.04) | +0.07  (0.05) |
| 75 mins |  |  |  | - | +0.04  (0.03) | +0.06  (0.04) | +0.06  (0.04) |
| 90 mins |  |  |  |  | - | +0.02  (0.03) | +0.02  (0.04) |
| 105 mins |  |  |  |  |  | - | -0.01  (0.03) |

**Supplementary Table 4.** Mean Difference (Standard Error) in Blink Duration for Post-Hoc Comparisons for Time-On-Task

Note: ***= P_adj_ <.001, **= P_adj_ <.010; *= P_adj_ <.050

|  | 30 mins | 45 mins | 60 mins | 75 mins | 90 mins | 105 mins | 120 mins |
| --- | --- | --- | --- | --- | --- | --- | --- |
| 15 mins | +0.52***  (0.11) | +0.32  (0.13 | +0.56**  (0.15) | +0.49*  (0.17) | +0.71**  (0.18) | +0.58*  (0.19) | +0.55*  (0.21) |
| 30 mins | - | -0.21*  (0.11) | +0.04  (0.13) | +0.04  (0.15) | +0.19  (0.17) | +0.06  (0.18) | +0.02  (0.19) |
| 45 mins |  | - | +0.25*  (0.11) | +0.17  (0.13) | +0.39*  (0.15) | +0.26  (0.17) | +0.23  (0.18) |
| 60 mins |  |  | - | -0.07  (0.13) | +0.15  (0.13) | +0.02  (0.15) | -0.02  (0.17) |
| 75 mins |  |  |  | - | +0.22*  (0.11) | +0.09  (0.13) | +0.06  (0.15) |
| 90 mins |  |  |  |  | - | -0.13  (0.11) | -0.16  (0.13) |
| 105 mins |  |  |  |  |  | - | -0.03  (0.11) |

**Supplementary Table 5.** Mean Difference (Standard Error) in Blink Rate/Min for Post-Hoc Comparisons for Time-On-Task.

Note: ***= P_adj_ <.001, **= P_adj_ <.010; *= P_adj_ <.050

|  | 30 mins | 45 mins | 60 mins | 75 mins | 90 mins | 105 mins | 120 mins |
| --- | --- | --- | --- | --- | --- | --- | --- |
| 15 mins | +3.7  (3.96) | +7.40  (4.54) | +10.69  (4.71) | +14.43*  (4.76) | +10.65  (4.75) | +17.23**  (4.67) | +15.11**  (4.40) |
| 30 mins | - | +3.66  (3.96) | +6.96  (4.54) | +10.70  (4.71) | +6.91  (4.76) | +13.50*  (4.75) | +11.38  (4.67) |
| 45 mins |  | - | +3.30  (3.96) | +7.04  (4.54) | +3.25  (4.71) | +9.83  (4.76) | +7.72  (4.75) |
| 60 mins |  |  | - | +3.74  (3.96) | -0.05  (4.54) | +6.54  (4.71) | +4.42  (4.76) |
| 75 mins |  |  |  | - | -3.79  (3.96) | +2.80  (4.54) | +0.68  (4.71) |
| 90 mins |  |  |  |  | - | +6.59  (3.96) | +4.47  (4.54) |
| 105 mins |  |  |  |  |  | - | -2.12  (3.96) |

**Supplementary Table 6.** Mean Difference (Standard Error) in Long Eye Closure Duration for Post-Hoc Comparisons for Time-On-Task.

Note: ***= P_adj_ <.001, **= P_adj_ <.010; *= P_adj_ <.050

|  | 30 mins | 45 mins | 60 mins | 75 mins | 90 mins | 105 mins | 120 mins |
| --- | --- | --- | --- | --- | --- | --- | --- |
| 15 mins | +0.25*  (0.10) | +0.41**  (0.12) | +0.56***  (0.12) | +0.66***  (0.13) | +0.55***  (0.13) | +0.75***  (0.13) | +0.65***  (0.13) |
| 30 mins | - | +0.16  (0.10) | +0.32*  (0.12) | +0.41**  (0.12) | +0.30*  (0.13) | +0.51***  (0.13) | +0.40**  (0.13) |
| 45 mins |  | - | +0.16  (0.10) | +0.25  (0.12) | +0.14  (0.12) | +0.35*  (0.13) | +0.25  (0.12) |
| 60 mins |  |  | - | +0.09  (0.10) | -0.02  (0.12) | +0.19  (0.12) | +0.09  (0.13) |
| 75 mins |  |  |  | - | -0.11  (0.10) | +0.10  (0.12) | -0.01  (0.12) |
| 90 mins |  |  |  |  | - | +0.20  (0.10) | +0.10  (0.12) |
| 105 mins |  |  |  |  |  | - | -0.10  (0.10) |

**Supplementary Table 7.** Mean Difference (Standard Error) in Long Eye Closure Rate/min for Post-Hoc Comparisons for Time-On-Task.

Note: ***= P_adj_ <.001, **= P_adj_ <.010; *= P_adj_ <.050

|  | 30 mins | 45 mins | 60 mins | 75 mins | 90 mins | 105 mins | 120 mins |
| --- | --- | --- | --- | --- | --- | --- | --- |
| 15 mins | +0.41***  (0.08) | +0.59***  (0.11) | +0.74***  (0.12) | +0.82***  (0.14) | +0.80***  (0.15) | +0.89***  (0.15) | +0.84***  (0.16) |
| 30 mins | - | +0.18*  (0.08) | +0.33**  (0.11) | +0.41**  (0.12) | +0.39*  (0.14) | +0.49**  (0.15) | +0.43*  (0.15) |
| 45 mins |  | - | +0.15  (0.08) | +0.23  (0.11) | +0.21  (0.12) | +0.31  (0.14) | +0.25  (0.15) |
| 60 mins |  |  | - | +0.08  (0.08) | +0.06  (0.11) | +0.16  (0.12) | +0.10  (0.14) |
| 75 mins |  |  |  | - | -0.02  (0.08) | +0.07  (0.11) | +0.02  (0.12) |
| 90 mins |  |  |  |  | - | +0.10  (0.08) | +0.04  (0.11) |
| 105 mins |  |  |  |  |  | - | -0.05  (0.08) |

**Supplementary Table 8.** Mean Difference (Standard Error) in PERCLOS for Post-Hoc Comparisons for Time-On-Task.

Note: ***= P_adj_ <.001, **= P_adj_ <.010; *= P_adj_ <.050

|  | 15 mins | 30 mins | 45 mins | 60 mins | 75 mins | 90 mins | 105 mins | 120 mins |
| --- | --- | --- | --- | --- | --- | --- | --- | --- |
| Pre-Drive | -0.30  (0.18) | +0.41  (0.22) | +0.54* (0.23) | +0.97***  (0.25) | +1.01***  (0.25) | +1.21***  (0.26) | +1.24***  (0.26) | +0.97**  (0.27) |
| 15 mins | - | +0.71***  (0.14) | +0.84***  (0.18) | +1.270***  (0.20) | +1.31***  (0.21) | +1.51***  (0.23) | +1.54***  (0.23) | +1.28***  (0.24) |
| 30 mins |  | - | +0.13  (0.13) | +0.557**  (0.17) | +0.60**  (0.19) | +0.79***  (0.21) | +0.82***  (0.21) | +0.56*  (0.23) |
| 45 mins |  |  | - | +0.43***  (0.11) | +0.47**  (0.15) | +0.66***  (0.17) | +0.69***  (0.18) | +0.43  (0.20) |
| 60 mins |  |  |  | - | +0.04  (0.11) | +0.24  (0.14) | +0.27  (0.16) | +0.01  (0.19) |
| 75 mins |  |  |  |  | - | +0.20*  (0.09) | +0.23  (0.11) | +0.04  (0.16) |
| 90 mins |  |  |  |  |  | - | +0.03  (0.08) | +0.23  (0.14) |
| 105 mins |  |  |  |  |  |  | - | +0.26  (0.12) |

**Supplementary Table 9.** Mean Difference (Standard Error) in KSS Scores for Post-Hoc Comparisons for Time-On-Task.

Note: ***= P_adj_ <.001 **= P_adj_ <.010; *= P_adj_ <.050

|  | 15 mins | 30 mins | 45 mins | 60 mins | 75 mins | 90 mins | 105 mins | 120 mins |
| --- | --- | --- | --- | --- | --- | --- | --- | --- |
| Pre-Drive | -0.07*  (0.03) | +0.02  (0.03) | +0.01  (0.04) | +0.13**  (0.04) | +0.10*  (0.04) | +0.14**  (0.04) | +0.11*  (0.04) | +0.10*  (0.04) |
| 15 mins | - | +0.09**  (0.03) | +0.09*  (0.03) | +0.20***  (0.04) | +0.18***  (0.04) | +0.22***  (0.04) | +0.18***  (0.04) | +0.17***  (0.04) |
| 30 mins |  | - | +0.01  (0.03) | +0.12**  (0.34) | +0.18*  (0.04) | +0.13**  (0.04) | +0.09*  (0.04) | +0.08  (0.04) |
| 45 mins |  |  | - | +0.12***  (0.03) | +0.09*  (0.03) | +0.13**  (0.04) | +0.10*  (0.04) | +0.01*  (0.04) |
| 60 mins |  |  |  | - | -0.03  (0.03) | +0.01  (0.03) | -0.03  (0.04) | -0.03  (0.04) |
| 75 mins |  |  |  |  | - | +0.04  (0.03) | +0.002  (0.03) | -0.003  (0.04) |
| 90 mins |  |  |  |  |  | - | -0.04  (0.03) | -0.04  (0.03) |
| 105 mins |  |  |  |  |  |  | - | -0.01  (0.03) |

**Supplementary Table 10.** Mean Difference (Standard Error) in LFA Scores for Post-Hoc Comparisons for Time-On-Task.

Note: ***= P_adj_ <.001, **= P_adj_ <.010; *= P_adj_ <.050
